# Supplementary material for: Content-rich biological network constructed by mining PubMed abstracts
Source: BMC Bioinformatics. 2004 Oct 8;5:147. doi: 10.1186/1471-2105-5-147 (PMC528731; doi:10.1186/1471-2105-5-147)
Supplement: Additional File 2 — The original results of the above study (non-essential files are deleted to keep the file size under the limit set by BMC bioinformatics). [file 1471-2105-5-147-S2.bz2 › chilibotAdditionalFile2/dip40/502ID7878020E10668/html/TGFB3_TGFBR2.html]

 


 **TGFB3** and **TGFBR2** 
  
Found 31 abstracts in PubMed, retrieved 31.  
 

 What does Google say? 
 PDF only 
| .edu only 

---

**Interactive relationship** (e.g. stimulation, inhibition, etc)

**Inhibitory relationship**- Whereas expression of TGF beta1, TGF beta2, and TGF beta type II receptor  [ **TGFBR2** ]  was suppressed by glucocorticoids in normal and wounded skin, expression of TGF beta3  [ **TGFB3** ]  and TGF beta receptor type I mRNA was stimulated.  Ref: 8626581 J Biol Chem, 1996
**Neutral relationship**- Furthermore, competition based quantitative reverse transcription PCR using synthetic internal standards revealed that leiomyomata express a significantly higher number copies per cell of TGF beta type II receptor  [ **TGFBR2** ]  mRNA, followed by TGF beta 1, TGF beta type I receptor, TGF beta 2, and TGF beta 3  [ **TGFB3** ]  P <.  Ref: 8784073 J Clin Endocrinol Metab, 1996
- In humans, TGF beta 3  [ **TGFB3** ]  is constitutively expressed in the epidermis of intact skin and in that of acute and chronic wounds a pattern of expression closely mirrored by the TGF beta type II receptor  [ **TGFBR2** ] .  Ref: 8277368 J Pathol, 1993

**Non-interactive relationship** (e.g. studied together, co-existance, homology, etc.)

- Human uterine tissue throughout the menstrual cycle expresses transforming growth factor beta 1, TGF beta 2, TGF beta 3  [ **TGFB3** ] , and TGF beta type II receptor  [ **TGFBR2** ]  messenger ribonucleic acid and protein and contains 125I TGF beta 1 binding sites.The use of isoform specific transforming growth factor beta primers, 35S labeled 40 mer oligonucleotide probes and polyclonal antibodies, reverse transcription polymerase chain reaction, in situ hybridization, and immunohistochemical observations has revealed that human uterine tissue at various reproductive stages expresses TGF beta s and TGF beta type II receptor  [ **TGFBR2** ]  messenger RNAs and proteins.  Ref: 8013382 Endocrinology, 1994
- In situ analysis of transforming growth factors beta TGF beta 1, TGF beta 2, TGF beta 3  [ **TGFB3** ]  and TGF beta type II receptor  [ **TGFBR2** ]  expression in basal cell carcinomas.  Ref: 8763422 Br J Dermatol, 1996
- Immunohistochemical localization of TGF beta type II receptor  [ **TGFBR2** ]  and TGF beta3  [ **TGFB3** ]  during palatogenesis in vivo and in vitro.  Ref: 9727838 Int J Dev Biol, 1998
- Messenger RNA was isolated from the nucleus pulposus using oligo 25 superparamagnetic beads and probing with gene specific primers in RT PCR. mRNA coding for TGF alpha, EGF, TGF beta1 and TGF beta3  [ **TGFB3** ]  or the EGF receptor and TGF beta type II receptor  [ **TGFBR2** ]  was found only occasionally.  Ref: 10615986 J Bone Joint Surg Br, 1999
- We therefore studied the expression of UPA, UPAR, IGF2R, ALK5,  **TGFBR2** , TGFBR3, ENG, ALK1, TGFB1, TGFB2, and  **TGFB3**  in a series of 14 pancreatic carcinoma cell lines.  Ref: 11408925 Int J Oncol, 2001
- In the present study, we have analysed and compared, by in situ hybridization, mRNA expression of transforming growth factors beta TGF beta 1, TGF beta 2, TGF beta 3  [ **TGFB3** ]  and TGF beta type II receptor  [ **TGFBR2** ]  T beta R II, and, by immunohistochemistry, the distribution of TGF beta 3  [ **TGFB3** ]  protein in normal human skin and in basal cell carcinoma BCC.  Ref: 8763422 Br J Dermatol, 1996
- The reverse transcription polymerase chain reaction revealed the predicted 44 1 24, and 431 basepair fragments for TGF beta 1, TGF beta 2, TGF beta 3  [ **TGFB3** ] , and TGF beta type II receptor  [ **TGFBR2** ] , respectively, in both endometrial and myometrial tissues, which were further verified by restriction enzyme analysis.  Ref: 8013382 Endocrinology, 1994
- In situ analysis of transforming growth factor beta s TGF beta 1, TGF beta 2, TGF beta 3  [ **TGFB3** ] , and TGF beta type II receptor  [ **TGFBR2** ]  expression in malignant melanoma.  Ref: 7614683 Carcinogenesis, 1995
- We demonstrate that primary cultured human thymic epithelial cells TEC express TGF beta 1, TGF beta 2 and TGF beta 3  [ **TGFB3** ] , as well as TGF beta type I receptor T beta RI ALK 5 and TGF beta type II receptor  [ **TGFBR2** ]  T beta RII transcripts.  Ref: 8562514 Int Immunol, 1995
- In this study we have investigated the expression of the precursor forms of TGF beta1, TGF beta2, TGF beta3  [ **TGFB3** ] , as well as TGF beta type I receptor TGF betaRI and TGF beta type II receptor  [ **TGFBR2** ]  TGF betaRII in the synovial tissue of arthritic rats during the course of the disease.  Ref: 9010265 Clin Exp Immunol, 1997
- Here, we present the 2.15 A crystal structure of the extracellular ligand binding domain of the human TGF beta type II receptor  [ **TGFBR2** ]  ecTbetaR2 in complex with human TGF beta3  [ **TGFB3** ] .  Ref: 11850637 Nat Struct Biol, 2002
- Corresponding increases in the levels of proliferating cell nuclear antigen PCNA and intracellular TGF beta 3  [ **TGFB3** ]  expression and a downregulation of the TGF beta type II receptor  [ **TGFBR2** ]  expression were also observed in the granulation tissue of the ulcer margins.  Ref: 9361170 Scand J Gastroenterol, 1997
- The present in situ hybridisation study describes expression of TGF beta 1, TGF beta 2, TGF beta 3  [ **TGFB3** ] , and TGF beta type II receptor  [ **TGFBR2** ]  mRNA during follicular development, and the temporal pattern and abundance of TGF beta 2 transcripts in early pre implantation embryos.  Ref: 8002998 Biochem Biophys Res Commun, 1994
- Expression of transforming growth factor beta isoforms and TGF beta type II receptor  [ **TGFBR2** ]  messenger ribonucleic acid and protein, and the effect of TGF beta s on endometrial stromal cell growth and protein degradation in vitro.Reverse transcription polymerase chain reaction analysis of total RNA and immunocytochemical observations revealed that human endometrial glandular epithelial and stromal cells in primary culture express messenger RNAs and proteins for transforming growth factor beta 1, TGF beta 2, and TGF beta 3  [ **TGFB3** ]  as well as TGF beta type II receptor  [ **TGFBR2** ] .  Ref: 8013384 Endocrinology, 1994
- We have analysed, by in situ hybridization, mRNA expression of TGF beta 1, TGF beta 2, TGF beta 3  [ **TGFB3** ] , and of TGF beta type II receptor  [ **TGFBR2** ]  in benign melanocytic naevi, primary melanomas, and in skin metastases of malignant melanomas.  Ref: 7614683 Carcinogenesis, 1995
- In this assay, the apparent dissociation constant of the TGF beta type II receptor  [ **TGFBR2** ]  ectodomain for TGF beta 3  [ **TGFB3** ]  was approximately 150 nM this value is approximately 1000 fold higher than that of the cell membrane receptor complex of living cells.  Ref: 8917430 Eur J Biochem, 1996
